# Meaningfulness and attachment: what dreams, psychosis and psychedelic states tell us about our need for connection

**DOI:** 10.3389/fpsyg.2024.1413111

**Published:** 2024-06-20

**Authors:** Lawrence Fischman

**Affiliations:** ^1^Department of Psychiatry, School of Medicine, Tufts University, Boston, MA, United States; ^2^Fluence, South Portland, ME, United States

**Keywords:** meaningfulness, attachment, dreams, psychosis/schizophrenia, psychedelic, narrative self, free energy principle, relational expectancies

## Abstract

The human need to find meaning in life and the human need for connection may be two sides of the same coin, a coin forged in the developmental crucible of attachment. Our need for meaningfulness can be traced to our developmental need for connection in the attachment relationship. The free energy principle dictates that in order to resist a natural tendency towards disorder self-organizing systems must generate models that predict the hidden causes of phenomenal experience. In other words, they must make sense of things. In both an evolutionary and ontogenetic sense, the narrative self develops as a model that makes sense of experience. However, the self-model skews the interpretation of experience towards that which is predictable, or already “known.” One may say it causes us to “take things personally.” Meaning is felt more acutely when defenses are compromised, when the narrative self is offline. This enables meaning-making that is less egocentrically motivated. Dreams, psychosis, and psychedelic states offer glimpses of how we make sense of things absent a coherent narrative self. This has implications for the way we understand such states, and lays bare the powerful reach of attachment in shaping what we experience as meaningful.

## Introduction

The closing line of Letheby and Gerrans’s (2017) much-cited paper, “Self Unbound” is a quotation from James Kingsland’s book, *Siddharta’s Brain*: “We have evolved into an ape that takes things personally” (Kingsland, 2016, p. 209). This provocative metaphor alludes to how humans model (make sense of) their phenomenal surroundings in ordinary states of waking consciousness by comparing their impressions with prior personal experiences. It refers directly to our use of predictive processing. This contrasts with how humans process their surroundings in non-ordinary states of consciousness, such as those induced by high doses of psychedelic drugs and in dreams. In framing their conclusion in an evolutionary context, Letheby and Gerrans invite speculation about the adaptative implications of our particular way of processing experience.

Philosophers, poets, and scientists have long noted similarities between our ways of thinking in dreams and madness, a short list to which, in the last century, psychedelic states have been added. The repeated finding in studies comparing cognition in people with and without schizophrenia that people with schizophrenia are less susceptible to visual illusions has been interpreted as suggesting a strength, rather than a deficit, of this style of thinking. What is significant about this assessment is not so much whether it truly represents a strength, which is debatable, but the idea that its adaptiveness is being considered at all. As discussed below in more detail, this finding illustrates a way in which (some) humans have evolved not to take things personally. The observation that people with schizophrenia are less susceptible to visual illusion may reflect an under weighting, or reduced attribution of precision, to prior experience. Ironically, by not allowing prior experience (expectation) to color perception, or by not, in Kingsland’s terms, taking things personally, persons with schizophrenia become susceptible to the idea of reference, the conviction that unrelated phenomena have personal relevance or meaning, which grows out of the primary delusional mood, the *sine qua non* of psychotic experience according to Jaspers (1913/1997; Mishara and Fusar-Poli, 2013).

This somewhat paradoxical observation calls for a careful examination of the processes which give rise to a sense of meaningfulness, and how the construction (and deconstruction) of narratives, including one’s sense of self, bear upon these processes. I will try to show how one’s sense of meaningfulness is related to one’s felt sense of connectedness with the attachment object. The infant exists continually on the brink of “impingements” which may induce feelings of annihilation: “Going to pieces. Falling forever. Having no relationship to the body. Having no orientation” (Winnicott, 1962); the infant’s sense of security rests upon feeling connected with the attachment object. The sense that the caregiver is emotionally invested in the infant’s well-being (motivated) and able to recognize her needs (capable) determines the infant’s sense of security within this relationship. As I will argue, the subject’s sense of connection with the caregiver in the attachment relationship (trust) models the subject’s sense of meaning in later life.

Narratives, particularly the self-narrative, which embodies both the sense of connection with the attachment object and defenses against awareness of the limits of that connection, create a sense of meaning. The unraveling of the narrative self that occurs in dreams, psychosis, and psychedelic states affords glimpses of un-relatedness, of meaninglessness, of annihilation. At the same time, these states afford the opportunity to form new narratives. In the transition, we can observe the process of meaning-making, of determining relatedness, or experiencing connection. It is a powerful affective process that during ordinary states of consciousness largely takes place unconsciously.

The loss of the narrative self in dreams, psychosis, and psychedelic states is experienced as an intense, complex, ambiguous, risk-filled, content-rich state; its affective valence ranges from highly pleasant to terrifying. In this presumably neuroplastic or critical state, the subject feels powerfully compelled to know how she relates to her surroundings. The subject’s usual repertoire of prior generative models is no longer accessible as a guide for allocating precision, yet the biologically driven exigency to ascertain causes persists. Here, the ape cannot take things personally. The process of meaning-making, no longer implicit, assumes a strongly felt, conscious quality^1^. The new relations experienced in such states carry greater significance, as if the subject somehow senses that she is no longer taking things personally.

This paper casts the process of meaning-making, which is amplified by the dissolution of the narrative self in dreams, psychosis, and psychedelic states, as driven by the same motivation—a biologically and psychologically determined need for connection—which turns the infant and her caregiver towards each other in the primary attachment relationship.

## Attachment

Bowlby describes attachment as a relational system of fitted infant and caregiver behaviors whose evolutionary purpose is to protect the infant from internally generated distress and externally perceived danger. The system builds upon genetically determined capacities for infant behaviors such as sucking, following, and clinging as well as for signaling internal states (e.g., crying and smiling), and coordinated motivated response capacities in the caregiver. Successful proximal interactions between infant and caregiver result in relieving the infant’s feelings of distress or perception of danger and instantiating a sense of security. They also condition the infant to seek proximity to the caregiver when feelings of distress or danger arise anew. When the infant experiences distress or danger in the absence of an attachment figure, separation anxiety ensues (Bowlby, 1960b). Prolonged unavailability of the caregiver engenders feelings of loss, leads to grief and mourning (Bowlby, 1960a).

Memories of attachment-based interactions with caregivers enable infants to develop representations or internal working models of these interactions, which are used to generate outcome predictions of future infant-caregiver interactions in a variety of circumstances and contexts. These models incorporate defenses used to mitigate distress resulting from insufficient caregiver responses. Caregiver responses are at least partly determined by the infant’s or child’s need activating the caregiver’s own early developed internal working models, which include their own characterological defenses. The behaviors resulting from the interaction between child and caregiver lead to consistent patterns of interaction that fall into categories that can be labelled as secure or insecure attachment, with the latter category subdivided into avoidant, ambivalent, or disorganized types. Essentially, these categories describe the nature and quality of connectedness in primary object relationships, which transposes to the quality of object relatedness in general.

The key points about attachment which will be developed further below are its role as the developmental template for the phenomenological experience of connection in later object relatedness, and its essential value in terms of individual adaptation and species survival.

## Attachment and meaning

What constitutes the sense of meaningfulness? Meaningfulness simply refers to the subjective sense of having ‘meaning’ (Klinger, 1977; Park, 2010), which is a bit harder to define. Baumeister defines meaning as “shared mental representations of possible relationships among things, events, and relationships… meaning *connects* things” (Baumeister, 1991, p. 15). Proulx and Inzlicht (2012) supplement this definition by discussing the human reaction to the absence of meaning. They cite Camus, who held in his only philosophical essay (Camus, 1942/2004) that people naturally organize their experiences into systems of relationships. “When these systems are undermined by contradictory experiences, he believed that a corresponding feeling of absurdity motivates efforts to make sense of experiences in other ways” (Proulx and Inzlicht, 2012). They also cite Kuhn, who argued that scientists naturally organize their observations into paradigms (Kuhn, 1962). “When these paradigms are violated by anomalous observations, Kuhn believed that the corresponding anxiety motivates efforts to make sense of the observations in other ways” (Proulx and Inzlicht, 2012). They cite Piaget, too, who theorized that cognitive development is built upon schemata, or organized representations of reality constructed from experience. When subsequent experiences fail to match our schemata, Piaget “believed that the corresponding disequilibrium motivates us to find other ways to make these experiences make sense” (Proulx and Inzlicht, 2012).

These and related observations prompted the meaning maintenance model (MMM) (Heine et al., 2006), at the core of which is “the proposition that humans are inexhaustible meaning makers” (p. 91). The model recognizes both the human compulsion to “construct mental representations of expected relationships between people, places, objects, and ideas” and their urgent need “to reconstruct a sense of meaning whenever their meaning frameworks are disrupted” (p. 90).

Are disruptions in meaningfulness existential crises? There are, of course, many kinds and levels of disruptions of meaningfulness. The relative levels of distress caused by the appearance of a black queen of hearts in a deck of cards (Bruner and Postman, 1949), an unfair ruling, child abuse, and genocide differ widely, yet all of these events violate our sense of the expected relations of things. Without compensatory efforts to “make sense” of such events, they leave us distraught. More consequential disruptions of meaning, when protracted or repeated, cause us to question the value of life. Studies of the MMM suggest that adaptation to disrupted meaning is fluid. One may compensate for disrupted meaning in one area of one’s life by deepening one’s sense of meaning in another area. While other explanations are possible, the “fluid compensation” model suggests that the diverse sources of disruption to our sense of meaning may have a common dynamic underpinning.

The authors of the MMM suggest that this common dynamic underpinning is the maintenance of expected relations themselves, and the sense of control over one’s life this affords. They cite the evolutionary advantage that the human capacity for making associations or connections between people, events and things confers, especially in discerning the intentions (expected behaviors) of fellow humans (Heine et al., 2006, p. 92). But there may be a deeper motivation: disruption to expected attachment relationships with caregivers is a direct threat to survival. The urgency with which we defend our sense of expected relations between things (meaning) derives from their affective matrix in the attachment relationship (Bowlby, 1969; Stern, 1985).

## Meaning-making and free energy

The MMM was formulated without reference to the free energy principle and predictive processing models of the brain, but there are striking convergences between them. The reason for outlining these parallels here is to buttress the notion that making meaning does not simply enrich subjective experience. It serves an adaptive function which is critical for survival.

The free energy principle (FEP) is a “mathematical formulation of how adaptive systems… resist a natural tendency to disorder” (Friston, 2010, p. 127). To maintain fragile physiological systems and sensory states within life-compatible bounds, self-organizing systems must resist surprise. As “free energy is an upper bound on surprise” (Friston, 2010, p. 128), minimizing free energy minimizes surprise. Importantly for our discussion of meaning-making, “minimizing surprise is the same as maximizing the sensory evidence for an agent’s existence” (Friston, 2010, p. 128).

The FEP views the brain as an inference machine that maximizes evidence for its existence by generating models that predict and explain its sensory inputs. These models are essentially beliefs about the probable causes of its sensory inputs. They are updated within a hierarchical nervous system that tests predicted belief outcomes against actual sensory inputs at each level of the system. The hierarchical structure of the system allows belief updating at each level to reduce “prediction error” (the difference between predicted sensory input based on prior belief and actual sensory input).

There are two strategies that an organism may use to minimize surprise, or reduce prediction error. One, called perceptual inference, is via learning, or updating generative models so that new models more closely fit incoming sensory data. The other, termed active inference, involves taking action to sample data which more closely conform with existing models. The two strategies may be viewed as either changing the model to fit the world, or changing the world to fit the model (Seth, 2013).

The MMM, while not a mathematical model, is built around similar dynamics and motivational underpinnings. The FEP holds that living organisms are primarily motivated by a *resistance to disorder* (italics in original; Friston, 2010, p. 127). The MMM grew out of a recognition that “humans strive to create and maintain order, certainty, and value in light of challenges and abruptions in their endeavors to do so” (Heine et al., 2006, p. 89). Under the FEP account humans, construct generative models that are best guesses as to causes of their interoceptive and sensory inputs. Under the MMM account, humans construct mental representations aimed at making sense of their internal and external environments. Disruptions in meaningfulness under MMM are treated as “expectancy violations” (Proulx et al., 2012), which are analogous to prediction errors under FEP. To reduce prediction error under FEP, the subject changes the model to fit the world, or else changes the world to fit the model (perceptual or active inference). Under MMM, the subject most often uses assimilation or accommodation to resolve expectancy violations. In language borrowed from Piaget (2000), the MMM describes how these processes work: “Experiences that are inconsistent with our schemata will arouse a sense of *disequilibrium*, which in turn motivates an *assimilation* of the experience so that it matches our schemata, or an *accommodation* of our schemata so that they account for the experience” (Proulx and Inzlicht, 2012, p. 325).

Finally, a critical axiom of the FEP is that minimizing surprise is the same as maximizing the sensory evidence for an agent’s existence. Extending our analogy with the MMM, this suggests that the process of making meaning (reducing expectancy violation, or by analogy, prediction error) maximizes evidence for the agent’s existence. This has interesting implications. An illustration would be: ‘In making sense of my experience, I feel more confident that I exist.’ The negative of this contingent relationship between making meaning and existing would be: ‘When I cannot make any sense of my experience, I doubt my own existence.’ In this way, the logic of the FEP offers an interesting perspective on how a sense of meaning supports continued existence, and conversely, how losing a sense of meaning coincides with suicidal thoughts or actions.

While making meaning, or reducing prediction error, does not always carry life or death consequences, it seems reasonable that evolution favors agents that can successfully resist disorder within their environmental niche. Because infants lack the physical capacity and repertoire of prior experience to use predictive processing effectively, their sensory states must be intuited by caregivers, who employ perceptual and active inference to help them resist disorder (Fotopoulou and Tsakiris, 2017). The two-person developmental origin of minimizing surprise explains, through the analogy between making meaning and minimizing surprise, how the sense of connection within the attachment relationship is the prototype for one’s sense of meaning later in life.

## Meaningfulness, narratives, and attachment

Narrative is how we knit disparate encounters with our environment into a meaningful form. As Adler et al. (2016) state, “narratives communicate not just the events that are central to the life of an individual but the meaning these events hold for the narrator.” The construction of narrative enables identity, an “inner story of the self that integrates the reconstructed past, perceived present, and anticipated future to provide a life with unity, purpose, and meaning” (McAdams, 1995, p. 365). Scholars from different disciplines generally agree that narratives have a beginning, a middle, and an (anticipated) end (Stern, 1992). This obtains for the narrative we call the self as well. In the FEP account, the self-narrative is a story of resisting disorder. Its beginnings, and its inevitable conclusion, are bounds upon the dramatic range of possibilities that comprise its middle. As we have seen, this middle is a story of finding, losing, and re-finding meaning.

The self-narrative begins within the matrix of attachment. As mentioned above, attachment interactions between infant and caregiver are remembered and represented as internal working models (Bowlby, 1969), or as representations of interactions with others that have become generalized (Stern, 1985), and serve as relationship expectancies against which new or imagined experiences with others are compared. The invariant qualities of the subjects and objects which comprise these representations are woven into narratives about oneself and others, and importantly, narratives about self with others (Moutoussis et al., 2014). The linking of thoughts, feelings, memories, desires, hopes and fears into a story with temporal continuity supplies an identity, a story of ‘me.’ A measure of coherence may be found “even amidst manifest arbitrariness, illogic, senselessness, and incoherence” (Freeman, 2010, p. 185), and “includes within itself all of the equivocations, contradictions, struggles and hidden messages that find expression in personal life” (Gallagher, 2000, p. 20).

Recent accounts of narratives as active inference within a predictive processing system have highlighted their adaptive and evolutionary value. By organizing and condensing cognitive and affective elements of prior experience into relational themes, narratives can represent multiple events efficiently and meaningfully, which makes them ideal candidates for generative models that can be used predictively. As the way that agents minimize prediction error is through actively sampling their environment, it is crucial that agents recognize their own role in altering the environment which is sampled, i.e., in causing deeply hidden states. In this way, the process of minimizing prediction error results in refining the model of the self as an actor in the system, or as Hohwy and Michael (2017) put it, “Self-modeling is thereby a process that can be described in causal terms, as first finessing a model of the self, and then engaging that model in action, leading to further finessing of the model, and more action” (p. 376). The self-model constructed and refined in this manner as the hidden cause of active states may be viewed as a narrative that unifies “regularities or plotlines at different, interlocked time scales. Such a theory or narrative can be seen as an answer to the question: which kind of agent am I?” (Hohwy and Michael, 2017, p. 370).

In contrast to other models of a narrative self, the active inference self-model is, in part, pre-linguistic and unconscious. It is a narrative account in the sense that it subsumes events under higher order behavioral “regularities” over longer time scales, and in minimizing prediction error, both “constrains our interpretation of those events” and “actively shapes itself over time to align with those higher-level regularities” (Hohwy and Michael, 2017, p. 370). By enabling “cultural learning,” active inference with attachment figures enables “further finessing” of the self-model. Infants recognize early on that other people are agents with intentions. Infants and young children apply agent models to the behavior of others in much the same way they apply agent models to their own actions, through the process of active inference. They represent the goals and strategies of others based upon their observed actions within environmental affordances, and structure their learning around these models, as is evident in their imitation of adults. By aligning with these intuited intentions, infants progressively conform self-models to the goals and strategies of the key objects in their world.

This is consonant with the model of natural pedagogy (Gergely and Csibra, 2005) as extended by Fonagy (1998) and colleagues in their model of epistemic trust, whereby “the very experience of having our subjectivity understood—of being mentalized—is a necessary trigger for us to be able to receive and learn from the social knowledge that has the potential to change our perception of ourselves and our social world” (Fonagy and Allison, 2014, p. 372).

By enabling cultural learning, active inference in interactions with others leads to better predictive models of the self (Hohwy and Michael, 2017). In the FEP, free energy can be formulated as complexity minus accuracy (Friston, 2010). Narratives enable agents to decrease model complexity or increase model accuracy. As Bouizegarene et al. (2020, p. 21) states, “Narratives enter the generative model as prior constructs or beliefs that either enable more accurate explanations for an unfolding scenario or provide a simpler account, thereby minimising the complexity of belief updating”. They further speculate that these properties of narrative constructs account for their evolutionary value: “the capacity to build and share narratives evolved largely because they provide group members with the ability to engage in cooperative action by avoiding ‘unexpected’ states of uncoordinated or contextually inappropriate action; i.e., ‘this is how we do things here (and not in other ways)’” (p. 19). This presumptive evolutionary role for narratives is consistent with positing meaning-making as an existential need.

But perhaps the strongest argument favoring an evolutionary role for finding meaning comes from applying the FEP to attachment. Hopkins (2016) notes that “basic expectations connected with biological imperatives like homeostasis are woven into the functioning of the brainstem.” Free energy resulting from deviations in prior homeostatic expectations (regarding hunger, thirst, pain and temperature regulation, etc.) necessitates action in the form of regulatory interoceptive and motor responses. Beginning in infancy, these responses are generated within “prototype emotions systems” outlined by Panksepp (1998), Watt and Panksepp (2009), and Watt (2012). These prototype emotional systems enable the infant to navigate the vagaries of the interpersonal world on which her survival depends.

Most of these systems are roughly divisible into those which promote social connection (LUST, PLAY, NURTURANCE, SEPARATION DISTRESS) and those which promote defense (RAGE and FEAR) (Watt, 2012). The motivational aims of these systems are often in direct opposition. As Watt puts it, “We cannot simultaneously reach out to give and receive love and affection, and at the same time protect ourselves from injury and disappointment” (Watt, 2012, p. 103). Such conflict often arises within the context of attachment, which affords the infant the behavioral means of satisfying homeostatic regulatory needs she cannot meet herself. Hopkins (2016) cites the Strange Situation (Ainsworth and Bell, 1970) as an example of conflict within the attachment setting. In this situation, in which an infant is exposed to repeated separation distress, the generative models of an insecurely attached infant “seize up, becoming incapable of predicting whether an optimal sensorimotor trajectory will approach or avoid their mothers.” Hopkins stresses that such seizing reflects overly-fitted, or excessively complex generative models of infant-caregiver interactions.

Video microanalysis of infant-caregiver interactions reveal the origins of this excessive complexity in patterns of repeated mismatches or misattunements between infant and caregiver. Essentially, this refers to expectancy violations within the infant-caregiver dyad, or in FEP terms, significant prediction error. When left unrepaired, this leads to infants not feeling known by or knowing their caregivers, insecure attachment, and predicts future difficulties with social connectedness (Tronick et al., 1998; Lyons-Ruth and Jacobvitz, 2008; Beebe et al., 2012a,b; Beebe and Lachmann, 2014) or dissociation (Liotti, 2006). Or as Watt (2012, p. 103) states, “Continued powerful activation of systems for organism defense, aggravated by the failure of social bonding and the promotion of separation distress may eventually contravene in a fundamental way our very capacities for social connection.”

This has significant adaptive and therefore evolutionary implications. Watt continues,

although prototype emotional systems for social connection were presumably selected because they enhanced homeostasis and the probability of successful procreation and survival, these systems for social connection became *independent motivational systems of enormous power in the mammalian and hominid lines.* The vicissitudes of attachment and our desire to secure connections and the rewards of social connection and intimacy color the entire emotional trajectory of a human life (italics in original; 2012, p. 104).

This notion, which emerges from studies of “affective neuroscience” (see also Panksepp, 1998; Davis and Montag, 2019), suggests an evolutionary basis for the claim that the process of meaning-making is driven by the same motivation—a biologically and psychologically determined need for connection—which turns the infant and her caregiver towards each other in the primary attachment relationship. We shall now explore the process of meaning-making, or the subjective sense of meaningfulness, where its dynamics are laid bare by the dissolution of the self-narrative in dreams, psychosis, and psychedelic states.

## Dreams

The above discussion of meaning-making has only confirmed Kingsland’s contention that we have evolved into an ape that takes things personally. One might argue from the above that the pursuit of meaningfulness and the construction of the narrative self (i.e., taking things personally) are parallel if not identical processes. The circular logic of the FEP suggests that our efforts to find meaning or to make sense of our world, to determine the probable hidden causes of our sensory data, are identical with the process of gathering evidence of our own existence, our models of ourselves. But what happens when our models of ourselves are deconstructed, when the “binding” necessary to assemble wholes from parts are taken offline?

Since Freud’s seminal work on dreams and the discovery of REM sleep (Aserinsky and Kleitman, 1953), much scientific and philosophical discussion about dreams has debated whether or not dreams are meaningful. This has ranged from Freud’s position, that dreams are the “royal road to a knowledge of the unconscious activities of the mind” (Freud, 1900, p. 608) to Hobson and McCarley’s (1977) activation-synthesis hypothesis which holds that dream images are little more than random neuronal discharges, brain impulses which hold no meaning. Why has this become such a fierce debate? Why do humans look to dreams for their meaning?

This may be related to differences in the quality of the experience of meaningfulness between ordinary and non-ordinary states of consciousness. During ordinary waking consciousness, the process of meaning-making is seldom urgent, and usually takes place automatically, without our attention. We are seldom aware of our attribution of meaning to experience. Yet this is not so while we dream. In dreams we are absorbed by suspense. Meanings are uncertain, inconstant, and clamor for clarification. We are rapt, anxious, exhilarated, thoroughly “entangled” (Ricoeur, 1992, p. 30; Pace-Schott, 2013). We are immersed in a spatiotemporal here-and now (Windt, 2010). We often awaken from dreams with a sense of incompleteness, wishing we could resume them to learn their conclusions, as if they were narratives with a determined outcome. But most dream experiences are fragmentary. Upon awakening, these fragments often seem so real that it takes a moment to realize they were only dreams. Yet they seem so significant that we want to remember their details so we can reflect on them when fully awake. We sense that the elements of a telling narrative are present, if only we could make sense of them.

The enhanced sense of meaningfulness we have in dreams may derive in part from feeling less defended while we sleep, more vulnerable (Windt, 2018). It is not hard to imagine why this should be so, as we lose awareness of our surroundings and cannot move voluntarily. But this only touches on our physical vulnerability. In dreams, one loses (most of) one’s phenomenal sense of connection with one’s body. With this, the ties between the dreamer’s (minimal) self—the “I” in the dream—and the (waking) narrative self-representation are notably loosened (Windt and Metzinger, 2007). We also lose connection with our interpersonal regulatory mechanisms and expectancies, our ways of ascertaining and validating who we are and how we belong in the world. Without these checks and balances on our generative models, and without the coherent self-narratives in which our defenses are subtly interwoven, we become immersed headlong in imagined scenarios (Windt, 2015) which evoke our deepest fears and wishes. In dreams we contact the subliminal fantasies in which some part of us dwells, a cauldron of repressed feelings, unexpressed desires, fears, hopes, buried memories; all of the veiled threats that we keep at bay while awake in order to interact effectively and efficiently with our environment.

It is precisely for these reasons that we instinctively regard dreams as meaningful, as revealing something of ourselves which we ordinarily deny access. It is as though we know we are creatures who hide unpleasant facts while we wake and visit these concealed truths while we sleep.

It is not that we are never similarly absorbed or vulnerable while awake. We can be carried away by stories, films, music, games, etc. What are we carried away from? Not by chance we answer with idioms like we ‘forget our selves’ or ‘lose our selves.’ In losing ourselves, we are carried closer to the kind of mentation we experience regularly when we sleep, when the connection with our narrative selves is detached, reinforced by the physiological attributes of the sleep state. In dreams, access to the reality testing processes which are partially suspended in daydreams and other versions of absorption, is even more limited (Gerrans, 2014) by the near complete exclusion of sensory input and motoric action from the process of active inference.

Coleridge’s (1817) famous description of absorption in a fiction as a “willing suspension of disbelief” (p. 208) may be apt here. The willing suspension of disbelief equates to a voluntary easing or suspension of our wired-in tendency to search for the hidden causes of our sensory data. As this hierarchical system is constructed around top-down generative models of self, it becomes understandable why, in describing absorption, we choose idioms that convey the process of being carried away from, or losing our selves. In suspending disbelief, we stop behaving, if only transiently, like apes that take things personally. Active inference continues, but attention, or precision-weighting is shifted towards interoceptive signals (Pianzola et al., 2021).

I offer the following to illustrate how the search for meaning in a dream is driven by the need for connection. Some friends and I had an email discussion of a recent newspaper article by a Stanford graduate student (Ball, 2022) about the cutthroat competition taking place on college campuses. The writer mentions the suicide of Katie Meyer, a Stanford undergraduate who was also a talented soccer goalkeeper. She died shortly after she received a disciplinary letter from the university administration. Her parents sued Stanford. Initially, I felt her parents were wrong to blame the administration for something that was likely considerably more complicated than a reaction to a letter. But after thinking further about Ball’s article, I realized that the Meyers lawsuit would focus needed attention upon student mental health. I wrote to my friends, “Ball asks, ‘What would it look like if we stopped more often to help others along in their course of study or to think through the course of life together?’ I think this is probably a good place to start….”

That night I dreamt I was engaged in some debate about the significance of Katie Meyer’s suicide. Katie Meyer was variously an adult, an infant, Black (she was Caucasian actually), and pregnant with Katie Meyer at the time of her death. Still at least half-asleep, I thought about what the infant Katie Meyer was able to accomplish in just two short weeks of life; how her action spoke in a way she could not represent in words, and compared this with what would have happened if had she thought about suicide, but decided against it. I was engaged in some discussion about this, though with whom was unclear. I argued that in choosing suicide, Katie may have had a vision of what her suicide would engender, of lasting systemic changes she might achieve. I felt badly that she would never know what her death achieved. I wondered whether this weakened my argument. When more fully awake, I gradually realized that Katie Meyer was not an infant, nor was she Black, nor pregnant with herself at the time she died. But I recognized that my dream might illustrate what I am discussing in this paper: how we turn to dreams for a sense of meaningfulness. In the dream, I portrayed Katie Meyer’s decision to commit suicide as an act which gave her life lasting positive meaning— that instead of feeling shamed, victimized, and angry about Stanford’s disciplinary action, she may have died thinking about how her death would prevent other deaths and miscarriages of justice (by altering the way college administrations handle disciplinary issues and larger issues of mental health on campus, including support for victims of sexual assault), and may have died feeling some positive self-regard for making the ultimate sacrifice.

Hopkins (2016) discusses how dreams use counterfactual thinking to reduce complexity and thereby minimize variational free energy, in keeping with the FEP (Hobson et al., 2014). This theory underscores the critical biological role of REM sleep and dreams in allowing nocturnal synaptic pruning and model updating, which enables more adaptive and efficient generative models for daytime predictive coding (Hobson and Friston, 2012). It is consistent with Revonsuo’s evolutionary theories of dreams as trial simulations of threat and social reality (Revonsuo, 2000; Revonsuo et al., 2015). Hopkins holds that complexity in dreams is generated by emotional “overfitting” and is reduced in dreams through condensation, symbolization and reconsolidation of emotionally charged memories evoked by challenging daytime events, as first outlined by Freud (1900). Machine learning simulations suggest that complexity reduction during sleep may facilitate “aha” moments and insight (new meaning) (Friston et al., 2017).

To illustrate, emotional overfitting (stress) arose in my dream from several sources: I felt pressured as the psychiatrist in the group to make a meaningful comment to my friends in our email discussion about mental health on campus; I was anxious that evening about a planned presentation to a group of psychotherapists/psychiatrists the following day of a paper I wrote, which upon re-reading seemed poorly written; thinking about Katie Meyer’s parents having no premonition of their daughter’s suicide despite talking with her shortly beforehand stirred up my worst nightmare as a psychiatrist—failing to recognize the risk of suicide in a patient; the threatening letter Meyer received from the Stanford administration stirred up troubling fears in my own life related to a letter from an administrative body; finally, I worried about making a cogent argument to an imagined audience for this paper about the meaningfulness of dreams.

All of this is condensed and counterfactually resolved by representing Katie Meyer in the dream as being *in utero* [a critical plank of Hobson and Friston’s (2012) virtual reality generator theory is that REM sleep and “protoconsciousness” begin *in utero*]; as Black (casting her death, like that of George Floyd, as igniting a vast process of meaningful social change); pregnant with Katie Meyer (thus emphasizing paradoxically her suicide as a way of creating another life for herself).

The vague audience in the dream subsumes my college friends, the students in my class, whoever I imagine might oversee my practice, and whoever I imagine might read this paper. Some of the dream’s meaning, as mentioned, directly reflects feelings stirred by Katie Meyer’s suicide, but its affective intensity, the reason I had the dream, emerges from feeling that my attachment to important objects was at stake. It has a quality of immediacy, a sense of meaningfulness, that waking consciousness does not impart. It condenses events with a common effective valence suppressed by the narrative self in waking life to minimize surprise.

## Psychosis

As with dreams, there is something in the nature of psychosis which invites questions of meaningfulness, of discerning within the unexpected juxtaposition of ideas, words, and actions in “madness” something which transcends reason. Many writers, including Kant, Hughlings Jackson, Wundt, and Freud have viewed madness as a waking dream (reviewed by Zarcone, 1979; Carhart-Harris, 2007). Hobson (1999) wrote, “We can therefore regard our own dreams as a proper basis for studying a normal process that becomes exaggerated in psychosis” (p. 22). In his short story, *Eleanora*, Poe (1845) wrote, “They who dream by day are cognizant of many things which escape those who dream only by night.” In the words of Theseus in *A Midsummer Night’s Dream*,

Lovers and madmen have such seething brains,

Such shaping fantasies, that apprehend

More than cool reason ever comprehends (Shakespeare, 1595-96/1974, 5.1.4).

Jaspers’s (1913/1997) concept of the delusional mood illuminates the phenomenology of meaning-making in psychosis. In this view, delusion formation is a way of making sense of an unaccountable change in one’s sense of reality, a new existential feeling (Ratcliffe, 2005) in which things suddenly seem unexpectedly significant, meaningful, though it is unclear how or why. “Patients feel uncanny and that there is something suspicious afoot. Everything gets a *new meaning*…. Something seems in the air which the patient cannot account for” (Jaspers 1913/1997, p. 98). And later, “‘I noticed particularly’ is the constant remark these patients make, though they cannot say why they take such particular note of things nor what it is they suspect. First they want to get it clear to themselves” (p. 100). Jaspers holds that this delusional atmosphere or mood engenders a “delusional experience of reality in which the environment offers *a world of new meanings*” (p. 99; emphasis in original in both quotes). He terms this experience the “primary delusion” and notes the great relief these new meanings bring to the patient.

This view has been affirmed by subsequent writers (Arieti, 1955/1974; Rycroft, 1962; Bowers and Freedman, 1966; Maher, 1974; Maher and Ross, 1984; Roberts, 1991, 1992), and has been incorporated into the aberrant salience theory of delusion formation (Kapur, 2003; Roiser et al., 2008; Mishara and Fusar-Poli, 2013; Howes and Nour, 2016; Hayashi et al., 2021).

Jaspers emphasizes throughout his tome the importance of distinguishing between understanding (*Verstehen*) and explanation (*Erklären*). In his view, primary delusion arises from an “alteration in the personality” (p. 105), something that “in its essence cannot be *understood*” (p. 105; emphasis added), even if it may be *explained* further in terms of abnormal perceptions, affective states, and bodily feelings, which he elaborates with great nuance and specificity.

Parnas (2013) takes issue with Jaspers’s ‘no further understanding’ claim. Parnas and Sass (2001) view delusional mood as a prodromal stage of schizophrenia marked by an alteration in one’s sense of presence. “The most prominent feature of altered presence in the pre-onset stages of schizophrenia is an unstable sense of the groundedness, fullness, or reality of the self and a frequent, intimately correlated feeling of alienation from the world. The patient feels that a profound change is *afflicting* him, but he cannot verbalize and pinpoint *what exactly* is changing” (p. 105). They regard this change as a pre-reflective alteration of consciousness which simultaneously disrupts one’s sense of being the subject of experience (ipseity) and one’s sense of embeddedness in the world (see also Ratcliffe, 2017), where “*Ipseity* refers to the experiential sense of being a vital and self-coinciding *subject* of experience or *first person perspective on the world*” (Sass and Parnas, 2003, p. 428; emphasis in original).

In aligning their work within the phenomenological tradition in psychiatry (including Jaspers), Sass, Parnas and colleagues express this disruption in ipseity as a dysfunction of minimal selfhood (Nelson et al., 2014). This formulation refines prevalent models of schizophrenic psychosis as a reflection of a disordered narrative self, recognizing that disruptions in minimal selfhood necessarily disrupt narrative self-models (Sass et al., 2011). In their ipseity-disturbance model, derived from empirical research using the highly specific Examination of Anomalous Self-Experience (EASE) scale (Parnas et al., 2005), Sass and Parnas add richly to our explanation (*Erklären*) of the psychotic process, but stop short of rendering the experiential contents of this change more accessible to empathic understanding (*Verstehen*). We are still left to wonder, along with the patient, what is “*afflicting*” him, “*what exactly*” is changing?

The fundamental change noted by Jaspers and by Parnas and Sass in their patients is well-captured in the sense of perplexity in the statement: “Something is going on; do tell me what on earth is going on” (Jaspers 1913/1997, p. 98). What is implied is that ‘I’ no longer trust ‘my’ impressions, either about what is going on within ‘me’ or ‘out there.’ Experience takes on an uncanny “lurking” character, as captured by Conrad (1958/2013) in his analogy between the schizophrenic *Trema* (analogous to Jaspers’s delusional mood) and walking through a forest at night. “It remains undefined. It is the lurking itself. In that area between what is visible and what is ‘behind’ the visible (e.g., the particular tree), what we call the background, where what we cannot grasp becomes uncanny” (p. 265). The simplest and most introspectively or empathically accessible way of describing what is changing is the patient’s felt *sense of certainty about things,* or *trust*. This may be a direct consequence of a confused sense of agency. This diminution of agency is experienced globally (thoughts, feelings, sensations, movement, etc.) “(*Patient* 21)-Things just happen to me now and I have no control over them. I do not seem to have the same say in things any more. At times I cannot even control what I want to think about” (McGhie and Chapman, 1961). The first-person account of psychosis literature is rife with similar examples (Fusar-Poli et al, 2022). To save space, I quote the conclusion of a review of this literature: “the multiform possible alterations in perception and cognition do lead rather reliably to the progressive loss of a sense of self and to the depersonalizing feeling of loss of control over one’s thoughts, feelings, and behaviors that characterize the onset of an acute schizophrenic psychosis” (Freedman, 1974).

A faulty sense of agency has long been cited as a possible source of delusions of influence and control in schizophrenia (Frith, 1987; Daprati et al., 1997; Fletcher and Frith, 2009; Jeannerod, 2009), and experimental evidence suggests it can distinguish prodromal psychosis as well (Hauser et al., 2010). This distorted sense of agency and its accompanying sense of passivity—and thereby vulnerability— may be a direct impetus for the need to establish meaning, partly to explain this startling loss itself, but more so, to restore a sense of connection. A socially dependent organism might tolerate a loss of connection as long as homeostasis can be maintained autonomously, but a perceived loss of agency poses an immediate and dire threat to autonomous functioning. Closer inspection of the phenomenal experience of “disrupted ipseity” helps explain why compensatory efforts are aimed at restoring a sense of connection.

## The intersubjective basis of the sense of reality

“Ipseity” refers to what it is like to be the subject of experience, or to have minimal selfhood (Zahavi and Parnas, 1998, pp. 689–691). It coincides with the ‘givenness’ of one’s experience as ‘mine.’ When ipseity is disrupted, experience no longer feels automatically ‘given’ or ‘mine.’

I think the main warning signal was my identity – the safety of knowing that I was an “I” – was starting to crumble. I became increasingly insecure about whether or not I really existed, or if I was only a character in the book or of being someone had made up. I was no longer certain of who was controlling my thoughts and actions; was it me, or was it someone else – the author maybe? I started feeling insecure about whether or not I was alive, really alive, because everything felt so empty and gray. In my diary I replaced “I” with “she” and after a while I started thinking like this as well: “She was walking to school. She was sad and wondering if she was going to die.” And someplace within me, something was questioning if “she” was still “me” (Lauveng, 2012, p. 4).

While Zahavi (2014) argues that minimal self is a “pre-condition” for intersubjective experience, that self and other are not “co-constituted,” other writers argue that minimal self experience is inseparable from intersubjective experience. Ratcliffe argues that “the most basic sense of self is indeed developmentally dependent upon interactions with other people. Furthermore, it is interpersonally sustained, and continues to depend upon other people” (2017, pp. 2–3). He maintains that “minimal self cannot be distinguished from an interpersonally constituted self, and that self-disturbances in schizophrenia are equally relational disturbances” (2017, p. 3).

Blankenburg describes something akin to disruption of ipseity in attributing the schizophrenic prodrome to a loss of natural self-evidence or common sense. In this account, “the healthiness of common sense rests on habituality. The natural self-evidence of everyday existence draws its nourishment from just such a habituality” (Blankenburg and Mishara, 2001, p. 309). Habit and common sense are derived from “the intersubjective constitution of the life-world” (2001, p. 310). Blankenburg holds that through intersubjectivity “what occurs or does not occur between the family members—becomes internalized in the patient and plays some role in how the presuppositions for cognition, judgment, and, also, the ability to encounter others develop” (2001, pp. 309–10). These intersubjectively acquired presuppositions serve as standards of comparison for evaluating new situations. In the development of psychosis, this basis for comparison is somehow lost. Blankenburg quotes a 20 year old female patient diagnosed with schizophrenia:

What is it that I am missing? It is something so small, but strange, it is something so important. It is impossible to live without it. I find that I no longer have footing in the world. I have lost a hold in regard to the simplest, everyday things. It seems that I lack a natural understanding for what is matter of course and obvious to others…. Every person knows how to behave, to take a direction, or to think something specific. The person’s taking action, humanity, ability to socialize...all these involve rules that the person follows. I am not able to recognize what these rules are. I am missing the basics.... It just does not work for me.... Each thing builds on the next.... I do not know what to call this.... It is not knowledge.... Every child knows these things! It is the kind of thing you just get naturally (pp. 307–08).

Blankenburg emphasizes that what this patient feels she is missing “is not *only* the knowledge of the naturally understood and matter-of-course things of everyday life, it is also the manner in which she understands things to be this way or that” (p. 308). The loss of common sense therefore refers to a global change in the structure of intentionality, in the way possibilities are conceived, not just a lack of social knowledge. Importantly, the intentional possibilities opened through common sense arise from its intersubjective developmental origins in a matrix of “‘basic trust’ as the foundation of the person’s relationship to world” (p. 310).

Blankenburg’s loss of common sense, then, as illustrated by the young woman quoted above, is another way of conceptualizing the development of a state of perplexity, of uncertain meanings and possibilities, that Sass and Parnas describe as disrupted ipseity. Fuchs (2015), like these writers, links this process to its intersubjective origins, but adds “embodiment” to theory of mind-based explanations of intersubjectivity. “Bodily behavior is expressive, intentional, and meaningful within its context…. It constitutes a sphere of primary intercorporeality as the basis for all forms of intersubjectivity” (pp. 193–94). In this “enactive” view, compatible with earlier discussed active inference models, “organisms do not passively receive information from their environment which they then translate into internal representations; rather, they constitute or enact the world through their sensorimotor interactions with the environment” (p. 204). Meanings are continuously “co-constructed” with others. “We live in a shared life-world because we continuously create and enact it through our coordinated activities and ‘participatory sense-making’” (p. 204). The rules governing these sense-making interactions are similar to those of Blankenburg’s common sense. Along with Blankenburg, Fuchs identifies “one most important element of this background” as “a basic sense of *trust*” (emphasis in original; p. 205). “This ‘bedrock’ of unquestioned certainties is a fundamental presupposition of the consensual understanding of a situation” (pp. 205–06).

Delusional mood results from the breakdown of this joint negotiation of meaning and basic trust in others. Fuchs therefore views delusions as a “relational phenomenon,” or a breakdown in “enacting a world through interaction with others” (p. 208). A similar explanation may account for the kind of perceptual changes which take place in schizophrenia, too. To explain this, Fuchs (2008) calls on Husserl’s theory of perception. Each time we look at an object (e.g., a table), we only see a part of it, one angle of, it as it were. Yet we make implicit assumptions about it as if the whole object were given to us. Perception “overcomes its own limited perspective by *intending* the object through its nonseen aspects” (p. 281). Rather than simply recording observable aspects of an object like a camera, perception actively constitutes an object by synthesizing many intentive processes, including temporal processes, which enable experiencing a table as the same table seen at different times. According to Husserl, this rests on the presumption that other subjects could perceive hidden aspects of the table from other angles.

In this way, Fuchs continues, “the Other is always already copresent in my habitual way of perceiving objects” (p. 282). Intersubjectivity is thus an integral part of the constitution of conscious (and unconscious) perception. The process of apprehending meanings (of gazes, actions, stories, etc.) rests upon the same process of synthesizing the imagined perspectives of other subjects, and allows the experience of meaningful unity. Rather than viewing someone as contracting hip flexors on one leg, lifting that leg off the ground, shifting one’s weight to the other leg, extending that leg at the knee, etc., one experiences these actions as “walking.” Fuchs (2008, p. 282) holds that this synthetic process is disturbed in schizophrenia, and that this disturbance may derive from the disruption in ipseity cited by Parnas and Sass.

How might this account for the altered perception Jaspers and others have described in the delusional mood? The habitual organizing principles that comprise common sense no longer apply. Removing intersubjectively derived (consensually validated or “known”) unifying meanings leaves an intentional void; perplexity and surprise reign. Salience, the relation of information to adaptive needs, can no longer be gauged by habitual associations or expectations. Everything feels significant, but without conventional prior meaning. Fuchs writes, “Single aspects or details of the perceptual field, now no longer framed and kept in distance by active intention, may become prominent, leap at the perceiving subject, catch him or penetrate into him. Especially the gaze of others, the quintessence of expression, obtains a captivating and piercing power” (p. 283).

## Linking the sense of reality to attachment

We will return to these circumstances later in discussing psychedelic states. In summarizing our discussion of psychosis thus far, we have seen that meaning is intersubjectively constituted; that its disruption leads to intolerable perplexity or uncertainty; that this creates an urgent search for meaning, and that new meaning, albeit delusional, affords great relief. The relational origins of meaning-making in intersubjective processes suggests that *disruptions of ipseity in the psychotic prodome may signify threats to one’s sense of connection (attachment), and that the quest to re-establish meaningfulness is at core a wish to restore connection*. This hypothesis does not minimize genetic or neurodevelopmental contributions to the development of schizophrenia or psychosis in general. It is intended only to address triggers of psychosis and suggest a context for treatment.^2^

Ratcliffe (2017) endorses the idea that the minimal self is interpersonally constituted. In Ratcliffe’s view, our experience of things is always influenced by how we imagine another person is, or could be, experiencing those things. “The kind of confidence we have in our own beliefs involves their rootedness in a consensus world; and the significance that attaches to things -what we anticipate from them- is shaped by relations with others” (p. 21). In Ratcliffe’s view, our common sense (borrowing Blankenburg’s notion), our pre-reflective awareness of how we belong in the world, is intersubjectively determined. “The ordinarily presupposed sense of confidence or certainty, in which the modalities of intentionality are rooted, implicates other people through and through” (p. 21).

The loss of common sense in the prodrome to schizophrenia signals a loss of the “*basic capacity for trust*” (emphasis in original; p. 19), a prerequisite for intersubjective experience. Recognizing a link between a loss of the capacity for trust and the disruption of ipseity in prodromic schizophrenia posited by Sass and Parnas, Ratcliffe suggests that, at least in certain cases, the onset of schizophrenia may be linked to trauma. He mentions an often overlooked historical detail in the well-known case of M. Sechehaye’s patient with schizophrenia, “Renee.” Renee mentions that she began having bouts of unreality around the “same period I learned my father had a mistress and that he made my mother cry. This revelation bowled me over because I had heard my mother say that if my father left her, she would kill herself” (Renee, 1951, p. 22). In her state of unreality, when she “looked at a chair or a jug, I thought not of their use or function – a jug not as something to hold water and milk, a chair not as something to sit in – but as having lost their names, their functions and their meanings; they became “things” and began to take on life, to exist” (pp. 55–56). Ratcliffe highlights a link between Renee’s feared loss of connection with her mother and her sense of unreality, but he might also have pointed out that Renee’s sense of reality returned dramatically when her therapist, M. Sechehaye, to whom Renee transferred many of her feelings and fears and to whom she referred as Mama, conducted a well-timed “symbolic realization.” She gave Renee an apple in a play-acting role mimicking breast-feeding that allowed Renee to feel that Mama loved her. Renee tells us that immediately afterwards,

“I realized that my perception of things had completely changed. Instead of infinite space, unreal, where everything was cut off, naked and isolated, I saw Reality, marvelous Reality, for the first time. The people we encountered were no longer automatons, phantoms, revolving around, gesticulating without meaning; they were men and women with their own individual characteristics their own individuality. It was the same with things. They were useful things, having sense, capable of giving pleasure” (pp. 105–06).

Following this, subsequent losses and reinstatements of Renee’s sense of reality could be reliably traced to her sense of connection or disconnection from Mama.

Of course, this is only one case, but it remains one of the best documented case studies in the literature on psychosis. It strongly suggests a causal link, if not an outright identity, between a sense of connection and a sense of meaningfulness. There is a rich, if somewhat neglected, body of literature which views psychosis as a meaning-making process (Arieti, 1955/1974; Bentall, 2013; Martindale and Summers, 2013; Charles, 2017; Garrett, 2019, [see footnote p. 10 for an expanded reference list]; Ruffalo, 2019; Tan, 2022), and its treatment a process of examining these meanings (Larsen, 2004; Attard et al., 2017). Studies also suggest, consistent with Renee’s treatment, that the key element in recovery from psychosis is establishing a trusting relationship with the therapist (Arieti, 1955/1974; Winnicott, 1971; Stanghellini and Lysaker, 2007; Hartley, 2011; Koehler et al., 2013; Charles, 2017; Marcus, 2017; Garrett, 2019; Ruffalo, 2019; Tan, 2022). Trust arises from a long-term commitment on the part of the therapist to grasp the narrative meaning (make sense) of the psychotic person’s hallucinatory and delusional (and other) experiences, very often by tracing them back to the phenomenal states discussed above: a loss of ipseity, a state of perplexity, diminished sense of agency, trust, meaningfulness, or pre-reflective common sense.

Or perhaps just as likely, it arises from making a long-term commitment, period (Hawley, 2014). This aligns with the idea that psychosis is an attempt to find new meaning when one can no longer trust anything, when (nearly) all connections stop making sense. Ogden (1980) describes “schizophrenic conflict” as “a tension between wishes to maintain a psychological state in which meaning can exist versus actual attacks on the capacities to create and maintain meaning” (p. 516). For “heuristic” purposes, he outlines four “stages” of this conflict, which include pre-psychotic and psychotic stages, and how these manifest in the therapeutic relationship through projective identification. In this in-depth case study, a person with schizophrenia (Phil) enters treatment in a state in which nothing has any meaning for him, all experience is devoid of affective value and therefore interchangeable; he does not even have a dysphoric sense of meaninglessness. He shows no engagement with the therapist. After a few months of regular sessions, he begins to project a sense of meaninglessness onto the therapist. Through projective identification, he begins to experience meaninglessness himself, but also begins to attach some meaning to things. This leads to a stage of overt psychosis (auditory hallucination, thought blocking and paranoia), but also to beginning to value his therapist. In this stage, overt psychosis is seen as a sign of progress towards resolving the “schizophrenic conflict” about experiencing meaning. This coincides with viewing the therapist “as a person who may be able to help with one’s frightening experiences” (p. 525). Finally, after 2 years of intensive therapy, Phil enters the stage of “symbolic thought. He says, “‘I’m able to think now. I could not before. Now that I can think, I can know what I’ve been thinking about’” (p. 527).

Ogden concludes, “In the course of the resolution of the schizophrenic conflict, the patient allows progressively more attention to, organization of, and attachment of meaning to his perceptions” (p. 528). In essence, Ogden sees schizophrenic psychosis as a manifestation of unconscious conflict between the wish to have meaningful experience and a protective need to limit the experience of meaningfulness. The balance of these conflicting forces is altered through gradual trust in the psychotherapeutic relationship via constructive projective identification, a process that involves both fantasied projection and real interpersonal interaction. He coins the term “actualization fantasy” in “an attempt to address the interface between the sphere of psychological meanings and representations (e.g., thoughts, motivations, and fantasies) and the sphere of the person’s capacities to create meanings and representations” (p. 530). The salient point Ogden calls attention to here is the *vital link in psychosis between the intersubjective and interpersonal interaction with the therapist and the capacity to experience meaning*.

## Models of psychosis and models of dreams

The previous section was meant to extend what Jaspers termed understanding (*Verstehen*) of delusional mood in a way which might allow a deeper dialog with someone who feels as if “something’s going on.” This section will focus on further explaining (*Erklären*) this feeling.

The main themes discussed above, losing one’s sense of meaning, connection, agency, control, and trust can all be related to expectancy. This bring us back to the interesting observations mentioned in the introduction, that there are some psychometric measures on which people with psychosis outperform “controls” (Dakin et al., 2005). There is a voluminous literature on the topic of resistance to visual illusions in people with schizophrenia. The interested reader is referred to reviews by Notredame et al. (2014), King et al. (2017), and Costa et al. (2023). It is evident that further research is needed to resolve some contradictory data and competing explanations, but there is robust agreement that people with schizophrenia are less susceptible than controls to visual illusions, most notably to the Müller-Lyer, Ebbinghaus, and hollow mask illusions. This resistance to illusions is not limited to visual illusions. For example, it also includes the tactile/proprioceptive-based force-matching illusion (Shergill et al., 2003, 2005). Several plausible explanatory models have been proposed, including Bayesian accounts (Corlett et al., 2009; Fletcher and Frith, 2009; Adams et al., 2013), circular inference models, (Jardri and Deneve, 2013; Notredame et al., 2014; Leptourgos et al., 2022), source monitoring deficits (Nelson et al., 2014; Cannon, 2015) and difficulty accessing personal experience as context (Hemsley, 2005). There are other explanations as well. Whether the emphasis is upon reduced precision of top-down prior beliefs or increased precision-weighting of bottom-up sensory signals, a common element in these models is that inference in psychosis is significantly less influenced by prior experience, or expectancy (Dima et al., 2009).

The accounts mentioned above all attempt to explain a *deficit* or *abnormality* in pre-psychotic or psychotic thinking, even though the data they analyze reflect a superior (more accurate) performance among schizophrenic subjects compared with controls. The presumption that perceptual experiences in people with schizophrenia are maladaptive has significant downstream consequences in the treatment setting, particularly with regard to establishing trust in the therapeutic relationship. For reasons too complex to discuss here, formulations which view psychosis as meaning-making (Johnstone et al., 2011; Basset et al., 2014) or adaptive (Scheepers et al., 2018; Lancellotta and Bortolotti, 2019) have had difficulty gaining traction within the larger mental health community, despite documented successful outcomes and “consumer” satisfaction. Seeing resistance to illusion as an adaptive response to ambiguity, one which allows a fresh take on conflict or challenging situations without canalized interpretations, is a necessary and empathic step toward helping people with psychosis establish the trust needed to (re-)access the world of intersubjectively shared meanings.

It is also a step toward de-pathologizing the process of meaning-making in psychosis (Johnstone et al., 2011). Seeing objects outside of their everyday contexts is exactly what takes place in dreams. Currently accepted dream theories—seeing REM sleep as critical for optimizing the brain’s generative model of the world (Hobson and Friston, 2012), or seeing dreams as rehearsals for threat perception and avoidance (Revonsuo, 2000) or simulations of social reality (Revonsuo et al., 2015)—emphasize the evolutionary or adaptive value of dreaming. Recognizing the parallel between meaning-making in dreams and the resistance to illusions in psychosis builds supports for the view that the meaning-making process in psychosis is an alternative mode of consciousness with certain adaptative advantages. This is not a radical claim that psychosis is desirable or healthy, but that as a meaning making process, it may enable vulnerable individuals to move through a pre-psychotic phase of untenable conflict, unbearable meaninglessness or anxious perplexity in the same way that dreams, even nightmares, may prepare people to cope with challenging fears or stressors.

Nielsen and Germain (2000) have suggested

the observation that interactive character imagery is virtually universal to dreaming could lead forthright to a theory of dreaming as simulation of attachment relationships. Attachment relationships (Bowlby, 1969) are also fundamental to survival and may have been as essential to threat mitigation as were the behavioral strategies of running from predators and disasters…. (S)uch a socio-emotional function for dreaming would still have clear adaptive significance for dreams occurring today.

## Psychedelics and the loss of expectancy

In this section, we will explore the process of meaning-making in psychedelic states, particularly those on the way to, during, and following ego dissolution. Here, meaning-making is tied to the loss of expectancy. This will extend the argument for the adaptive value of meaning-making in dreams and psychosis, as well as the argument that meaningfulness represents a symbolic satisfaction of basic human attachment needs. Put slightly differently, the compelling human quest for meaning derives from evolutionarily programmed developmental needs for connection in order to assure safety and survival.

The meaning-enhancing properties of psychedelics have long been recognized (see Hartogsohn, 2018 for a summary; Cohen, 1964; Griffiths et al., 2006; Belser et al., 2017; Preller et al., 2017; Barrett et al., 2018; Miceli McMillan, 2020). Subjects often describe this as a direct perceptual experience. On mescaline, Huxley (1954) witnessed “a bunch of flowers shining with their own inner light and all but quivering under the pressure of the significance with which they were charged” (p. 5). Ordinary books on shelves became “lapis lazuli books whose color was so intense, so intrinsically meaningful, that they seemed to be on the point of leaving the shelves to thrust themselves more insistently on my attention” (p. 5). On mescaline, “The mind does its Perceiving in terms of intensity of existence, profundity of significance” (p. 5).

This new expanded significance, or salience, coincides with unbinding the self-representation (Letheby and Gerrans, 2017), or what is often called ego dissolution. The directly felt salience of objects during psychedelic-induced ego dissolution is phenomenologically similar to that experienced in psychosis. Matussek (1952/1987) writes (of psychosis), “Abnormal significance is perceptually encountered as an integral part of the object. It is not primarily deduced, thought out, or dredged up in some other way from our thoughts, but rather experienced directly as inherent in the object” (p. 98). These and similar observations suggest that the process of meaning-making in both states is altered from that which takes place during ordinary waking consciousness. Normally, meaning is apprehended as an outgrowth of perceived relationships or connections between things (Baumeister, 1991), and is intersubjectively and culturally influenced by the meanings attributed to the object or event by others. In psychedelic ego dissolution and psychosis, meaning is felt to inhere in the object; one feels as if in touch with the object’s “Is-ness” (Huxley, 1954, p. 5). In the predictive processing model, the sense of directly felt expanded significance or meaning may represent the phenomenological embodiment of a shift towards increased precision of “bottom-up” signaling relative to “top-down” generative modeling (Millière et al., 2018; Timmermann et al., 2018). This means sensory input is no longer (as) constrained by prior expectation (Swanson, 2018, p. 16). As Letheby and Gerrans eloquently state, “Attention is no longer guided exclusively by adaptive and egocentric goals and agendas; salience attribution is no longer bound to personal concern” (Letheby and Gerrans, 2017, p. 6). As this model would predict, subjects on LSD show the same resistance to the hollow mask illusion as people with schizophrenia (Passie, T., unpublished study reported in Caragol, 2009).

In a psychodynamic model, the sense of expanded significance reflects the loss of defense mechanisms which would ordinarily modulate affective intensity. This could account for the unaccustomedly powerful emotions one feels during ego dissolution, as well as the appearance of unitive feelings. As psychedelics “relax” the precision of prior expectations (Carhart-Harris and Friston, 2019), or “unbind” multisensory integration from models of self (Klee, 1963; Letheby and Gerrans, 2017) — really, models of self-with-other — then cautionary (defended) approaches to object relations based upon historically-derived expectations of frustration, disappointment, misattunement, trauma etc. no longer guide action policy.

This has important implications for attachment and feeling connected. Whether explained psychodynamically as a suspension of defensiveness, or computationally as a reduced precision of hierarchically-generated models of self(−with-other), psychedelics consistently foster feelings of increased connectedness with others, and more globally, unitive feelings, or feelings of oceanic boundlessness (Belser et al., 2017; Cosimano, 2017; Watts et al., 2017; Carhart-Harris et al., 2018; Kałużna et al., 2022). As discussed above, one’s sense of meaningfulness is closely tied to recognizing how things are connected or related. It is not surprising, therefore, that many people consider the unitive feelings they experience on psychedelics to be among the most meaningful experiences of their lives (Griffiths et al., 2006, 2008).

As stated above, the process of meaning-making in psychedelic-induced ego dissolution is tied to the drug’s interference with one’s capacity to attend selectively to personally relevant information, and to integrate information into narrative models of self-with other. This may be seen in the early stages of psychedelic-induced ego dissolution, where a loss of ipseity strikingly similar to that which presages the onset of psychosis occurs. In both, one’s implicit expectancies in relation to internal states is disturbed, and previously unconscious somatic and interoceptive sensations unwillingly become a focus of attention. “The entire body is felt; instead of being taken for granted, it becomes the entire focus of attention. Things that ordinarily occur out of awareness come into the foreground: the fillings in one’s teeth, the contractions of the stomach, and the urinary and anal sphincters, the pulsations of the arteries, the pounding of the heart, the sensations of the skin and muscles” (Savage, 1955, p. 7). The smooth concatenation of expectancies regarding self-states required to enable efficient allocation of attentional resources “to egocentric goals and agendas,” to function “like an ape that takes things personally,” is disturbed. The meaning-making process is disrupted

## Attachment and expectancy

Attachment is predicated upon generating expectancies of one’s own and one’s caregiver’s states and actions. Video microanalysis of mother–infant interaction shows that contingency-based expectancies at age 3–4 months regulate interpersonal behavior and predict the quality of attachment behavior at 12 months (Beebe et al., 2010). Contingency detection means anticipating how each partner’s actions affect oneself and one’s partner (Haith et al., 1988). Infants detect and respond to contingencies from birth (Papousek and Papousek, 1975; DeCasper and Carstens, 1981). Remembering these contingencies enables the construction of predictive sensorimotor schemas or internalized models of being with others (Bowlby, 1969; Stern, 1985). These schemas are the basis for implicit relational knowing (Stern, 1992; Lyons-Ruth et al, 1998, Lyons-Ruth, 1999), or knowing and being known (Beebe et al., 2012a,b; Beebe and Lachmann, 2014).

The Still-Face experiment, (Tronick et al., 1978) demonstrates how the disruption of expectancy, at a basic level, is a catastrophic threat to secure attachment. This frames the evolutionary need for strategies to adapt to expectancy disruptions. Yet secure attachment does not require highly contingent responses. In fact, “mid-range” maternal contingent responses turn out to be optimal for secure attachment (Beebe et al., 2012a). This is because discrepancies from expected interactions enable learning and adaptation, or updating of generative models, through important disruption and repair processes (Tronick, 1989; Tronick and Cohn, 1989; Beebe and Lachmann, 1994; Lachmann and Beebe, 1996). Expectancy disruption is, by definition, a disruption of known relations between things, a disruption of meaning systems. This directly links the process of finding meaning with the process of disruption and repair necessary to establish secure attachment.

Disrupting expectation is what enables the sense of expanded significance or meaningfulness under psychedelics (Fischman, 2019), and what allows dream content to unfold outside the rules of causation and logic. Together, the parallel between psychedelic states and dreams (Kraehenmann, 2017) and the critical role of expectancy disruption and repair in establishing secure attachment supports Nielsen and Germain’s (2000) theory mentioned above that dreams are simulations of attachment relationships and therefore have adaptive significance.

## Conclusion: meaningfulness and connection

The capacities to feel life as meaningful and to feel human connection have a common root in attachment relationships. Secure attachment affords a needed base from which one ventures out to discover new meanings in the world. It is no coincidence that the language we use to define ‘meaning’ — “shared mental representations of possible relationships among things, events, and relationships… meaning *connects* things” (Baumeister) — is the language we use to describe bonds between people. Psychodynamically, understanding someone or something affords peace of mind because it re-evokes the security of attachment. Our motivation to find meaning in life derives from our evolutionarily programmed need for proximity and support.

In disrupting our self-models, dreams, psychosis, and psychedelic states each serve as windows on the process of making connections between things. Each affords an opportunity to create meaning outside of the usual routes traversed by expectancy. It behooves us to regard the capacity to find meaning unbiased by egocentric agendas as useful, perhaps even evolutionarily vital.

Such an approach is inherent in theories and intuitions which see dreams as “the royal road to a knowledge of the unconscious activities of the mind” (Freud, 1900, p, 608); as “protoconsciousness” (Hobson and Friston, 2012); or as evolutionarily necessary simulations of threat or social reality (Revonsuo, 2000; Revonsuo et al., 2015). It is implied in therapeutic formulations which see psychosis as an individual’s struggle to make sense of a life that has lost meaning (Johnstone et al., 2011; Basset et al., 2014; Friesen, 2022) and which emphasize the critical role of the informed and committed psychotherapist in rebuilding an individual’s sense of trust. It is also present in approaching psychedelic-assisted psychotherapy as an opportunity to gain new perspectives and to experience first-hand that what we generally accept as “reality” is only a model of what we expect to see.

It is re-assuring to know that we do not always have to take things personally.

For now we see only a reflection as in a mirror; then we shall see face to face. Now I know in part; then I shall know fully, even as I am fully known.^3^

## Data availability statement

The original contributions presented in the study are included in the article/supplementary material, further inquiries can be directed to the corresponding author.

## Author contributions

LF: Writing – review & editing, Writing – original draft.

## Footnotes

^1^Less conscious, of course, in dreams.

^2^All of the authors whose studies of psychosis are discussed and/or cited in this section specifically make a similar acknowledgement.

^3^From 1 Corinthians 13:12, New International Version.
