# Erratum: Meaningfulness and attachment: what dreams, psychosis and psychedelic states tell us about our need for connection

**DOI:** 10.3389/fpsyg.2024.1509670

**Published:** 2024-10-24

**Authors:** Frontiers Production Office

**Affiliations:** Frontiers Media SA, Lausanne, Switzerland

**Keywords:** meaningfulness, attachment, dreams, psychosis/schizophrenia, psychedelic, narrative self, free energy principle, relational expectancies

Due to a production error, a portion of the text was omitted from the published article. In the section “The intersubjective basis of the sense of reality,” the first sentence of paragraph three was incorrectly written as “Blankenburg and Mishara (2001, p. 309).” This sentence should instead be written as shown below.

“Blankenburg describes something akin to disruption of ipseity in attributing the schizophrenic prodrome to a loss of natural self-evidence or common sense. In this account, “the healthiness of common sense rests on habituality. The natural self-evidence of everyday existence draws its nourishment from just such a habituality” (Blankenburg and Mishara, 2001, p. 309).”

The publisher apologizes for this mistake.

The original version of this article has been updated.

